# Risk Factor Levels and the Burden of Skin Melanoma in Poland with Predictions Regarding the 2020–2030 Perspective

**DOI:** 10.3390/jcm15124673

**Published:** 2026-06-16

**Authors:** Sławomir Porada, Aleksandra Czerw, Grażyna Dykowska, Natalia Czerw, Olga Partyka, Monika Pajewska, Tomasz Banaś, Izabela Gąska, Elżbieta Kaczmar, Katarzyna Sygit, Marian Sygit, Paulina Wojtyła-Buciora, Jarosław Drobnik, Piotr Pobrotyn, Dorota Waśko-Czopnik, Tomasz Sowiński, Katarzyna Tejza, Wojciech Homola, Łukasz Strzępek, Mateusz Curyło, Monika Urbaniak, Marcin Mikos, Elżbieta Grochans, Anna M. Cybulska, Daria Schneider-Matyka, Kamila Rachubińska, Ewa Bandurska, Weronika Ciećko, Barbara Majer-Giernat, Karolina Kamecka, Remigiusz Kozlowski

**Affiliations:** 1 Faculty of Health Sciences and Psychology, Collegium Medicum, University of Rzeszów, 35-310 Rzeszow, Poland; 2 Department of Health Economics and Insurance, Medical University of Warsaw, 00-581 Warsaw, Poland; 3 Department of Epidemiology, Maria Sklodowska-Curie National Research Institute of Oncology—Krakow Branch, 31-115 Krakow, Poland; 4 Students’ Scientific Organization of Cancer Cell Biology, Department of Oncology Propaedeutics, Medical University of Warsaw, 01-445 Warsaw, Poland; 5 Department of Radiotherapy, Maria Sklodowska-Curie National Research Institute of Oncology—Krakow Branch, 31-115 Krakow, Poland; 6 Medical Institute, Jan Grodek State University in Sanok, 38-500 Sanok, Poland; 7 Faculty of Medicine and Health Sciences, University of Kalisz, 62-800 Kalisz, Poland; 8 Department of Family Medicine, Faculty of Medicine, Wroclaw Medical University, 50-367 Wroclaw, Poland; 9 Department of Clinical Neurosciences, Faculty of Medicine, Wroclaw University of Science and Technology, 50-370 Wroclaw, Poland; 10 Department of Gastroenterology, Hepatology with Inflammatory Bowel Disease Subunit, Provincial Specialist Hospital J. Gromkowskiego, 51-149 Wroclaw, Poland; 11 Endocare Medical Center, Simple Joint-Stock Company (S.J.S.C.), 50-558 Wroclaw, Poland; 12 FemiMea Centre for Obstetrics and Gynaecology, 55-040 Bielany Wroclawskie, Poland; 13 Clinical Department of General, and Oncological Surgery, Saint Raphael Hospital, 30-693 Krakow, Poland; 14 Department of Surgery, Andrzej Frycz Modrzewski Krakow University, 30-705 Krakow, Poland; 15 Institute of Health Sciences, University of the National Education Commission, 30-084 Krakow, Poland; 16 Rehabilitation Department, Hospital of the Ministry of Interior and Administration in Krakow, 30-053 Krakow, Poland; 17 Department of Medical and Pharmaceutical Law, Faculty of Medicine, University of Medical Sciences, 61-701 Poznan, Poland; 18 Department of Bioinformatics and Public Health, Andrzej Frycz Modrzewski Krakow University, 30-705 Krakow, Poland; 19 Department of Nursing, Faculty of Health Sciences, Pomeranian Medical University in Szczecin, 70-204 Szczecin, Poland; 20 Center for Competence Development, Integrated Care, and e-Health, Medical University of Gdansk, 80-204 Gdansk, Poland; 21 Centre for Security Technologies in Logistics, Faculty of Management, University of Lodz, 90-237 Lodz, Poland; 22 Department of Management and Logistics in Healthcare, Medical University of Lodz, 90-419 Lodz, Poland

**Keywords:** cancer, melanoma, prevalence, epidemiology

## Abstract

**Background/Objectives:** Melanoma is a major and growing public health concern in Poland, with a five-year survival around 60–70%. While UV radiation and genetic susceptibility are well-known risk factors, lifestyle and environmental exposures may also contribute. This study examined how selected risk factors relate to one-year melanoma prevalence across Poland’s 16 voivodeships and assessed whether these factors can support short-term prediction. **Methods:** Annual melanoma prevalence for 2011–2021 was obtained from the Polish National Cancer Registry, and voivodeship-level estimates of metabolic risk factors, physical inactivity, alcohol consumption, smoking, high BMI, air pollution, water pollution and limited data on UV exposure were used to build a general estimating equations model. Model predictions for 2020–2021 were compared with observed data, and forecasts were generated through 2030. **Results:** Melanoma cases increased in every voivodeship between 2011 and 2021. Metabolic risk factors, high BMI, low physical activity and smoking were associated with higher melanoma prevalence. When other factors were considered, air pollution showed an inverse association, suggesting complex relationships that warrant further analysis. Forecasts indicated increasing prevalence in all of 16 voivodeships through 2030, although three regions showed large prediction errors for 2020–2021. A key limitation was the lack of sufficient UV exposure data. **Conclusions:** The findings support further evaluation of public health actions targeting the reduction of unhealthy lifestyle regarding diet, low physical activity, and smoking to help slow the projected rise in melanoma.

## 1. Introduction

The World Health Organization reports the incidence of melanoma to be equal to 331,722 cases worldwide in 2022 [1]. The age-standardised incidence rate is equal to 3.2/100,000, which makes it the 17th most prevalent malignancy. The incidence rate values for Europe and North America were higher, equal to 10.4/100,000 and 16.3/100,000, respectively, and for Oceania, the figure was 29.8/100,000 [1,2]. The highest incidence rate was recorded in Australia, with a value of 37.0/100,000. For Poland, 3751 cases in 2022 and an age-standardised incidence rate equal to 4.7/100,000 were reported.

Regarding gender, the incidence rates were higher in males than in females [1,2]. In the total world population, they were equal to 3.7/100,000 and 2.9/100,000 for males and females, respectively. In North America, rates were 19.0/100,000 and 14.3/100,000; in Oceania, 37.4/100,000 and 23.1/100,000; in Europe 11.0/100,000 and 10.1/100,000; and in Asia, 0.44/100,000 and 0.40/100,000 for males and females, respectively. For Poland, the incidence rate for males was equal to 5.4/100,000 and for females to 4.1/100,000. Therefore, in Europe, the incidence rates for males and females were closer to each other; however, in Poland, they differ.

The reported risk of developing melanoma based on data from the European Commission by age 74 is 1:74 for women and 1:66 for men [2]. The likelihood of five-year survival is high, typically ranging from 80% to 90%, for most European countries. In some countries, i.e., Slovakia, Croatia, Estonia, and Lithuania, the percentage is lower (70–80%); in Poland, it falls between 60% and 70%. In Bulgaria, it is equal to 50% [2].

Regarding mortality, melanoma is 22nd on the list of malignancy-related deaths. The global age-standardised mortality was equal to 0.53/100,000 [1]. Mortality rates were higher for North America at 1.1/100,000, Europe at 1.5/100,000, and Oceania at 2.3/100,000. In Poland, the age-standardised mortality rate in 2022 was equal to 1.9/100,00. The mortality rates were also higher in males than in females. In North America, they were equal to 1.5/100,000 and 0.74/100,000; in Oceania to 3.0/100,000 and 1.7/100,000; in Europe to 1.9/100,000 and 1.2/100,000; and in Asia to 0.24/100,000 and 0.19/100,000 for males and females, respectively. In Poland, the age-standardised mortality rate in 2022 was equal to 2.6/100,000 for males and to 1.4/100,000 for females. Therefore, the differences between females and males were larger in Poland than in Europe overall. In time, mortality rates decreased and incidence rates increased, which can be attributed to both better detection methods and higher intensity of risk factors related to lifestyle [3].

The main risk factors for melanoma are exposure to ultraviolet radiation from sunlight; tanning bed lamps; sunburn during childhood; having multiple (>100) pigmented nevi on the skin; presence of atypical nevi; melanoma or skin cancer in the family history; a melanoma diagnosed previously; other malignancy in medical history, especially thyroid cancer, soft tissue cancer, cervical cancer, and acute myeloid leukemia; high lifetime intake of alcohol [4]; a weakened immune system as a result of HIV infection, transplant, Non-Hodgkin’s lymphoma or chronic lymphocytic leukemia, for example; older age; fair complexion [5]; light eye color; freckles; skin phototype II according to the Fitzpatrick scale (which applies to skin that burns easily and is minimally susceptible to tanning) and skin type I and III when compared to IV. Regarding genetics, CDKN2A, CDK4, BAP1, MITF, TERT, ACD, TERF2IP or POT1 mutations and one or more variants of MC1R are a risk, as is having blonde or red hair [6]. The risk of melanoma is also higher among patients with xeroderma pigmentosum (XP) [7].

Among other risk factors, metabolic abnormalities, like insulin resistance, dyslipidemia, and hyperinsulinemia are mentioned [8]. Higher BMI is associated with an increased risk of developing both melanoma and non-melanoma skin cancers as well [9].

An increased level of physical activity is also demonstrated to be associated with a reduced risk of developing malignant melanoma [10], which suggests that low physical activity may be linked to an increased risk of melanoma. An investigation regarding the association between tobacco smoking and melanoma yielded conflicting results. Smoking was found to be an independent risk factor for melanoma, particularly in older individuals aged 60 years and above [11], but a negative relationship between smoking and melanoma risk was found, suggesting that smokers may even have a lower risk of developing melanoma compared to non-smokers [12]. Environmental pollutants, including industrial and chemical exposures, and pollutants released into the air are additional risk factors that may increase the incidence of melanoma and other skin cancers [13]. Chemical contaminants in water, specifically environmental pollutants, such as arsenic, were also found to be related positively to melanoma incidence [14].

The impact of risk factors can be measured in terms of disability-adjusted life years (DALYs), which is a measure combining both premature death and disability resulting from a disease [15]. Its components are the number of deaths multiplied by the standard life expectancy at the age of death and the number of prevalent cases multiplied by disability weight, which ranges from 0 to 1. One DALY equals one lost year of a healthy life. The values for DALYs were established empirically on the basis of the Global Burden of Disease data acquired from the Institute for Health Metrics and Evaluation for the period 1991–2023. It is the only comprehensive database providing estimates of risk factors for each voivodeship in Poland.

One-year prevalence of a disease is the number of patients who exhibit a given medical condition during a year. It is a crucial indicator for the medical care system, allowing for assessment of the dynamics of change in the prevalence of a disease on a year-to-year basis. It is usually reported as the ratio of the number of cases to each 100,000 people.

The purpose of the current paper is to assess the relationships between the estimates of risk factors and the estimates of one-year prevalence of melanoma on the basis of the data for Polish voivodeships and to evaluate the accuracy of a prediction model in order to assess the burden of melanoma in Poland and its dynamics.

## 2. Materials and Methods

Poland is administratively divided into 16 voivodeships. The estimates of one-year prevalence of melanoma in each of the 16 voivodeships in 2011–2021 were acquired from the Polish National Cancer Registry [16]. This is a population-based registry collecting data on new cancer cases in Poland. The range of collected data follows the guidelines of international organizations of cancer registries, which are in use at the World Health Organization and the European Commission. It allows for studying cancer epidemiology at the national and voivodeship level.

The share of risk factors in 2011–2019 in Poland was acquired from the System Analysis and Implementation Database provided by the Polish Ministry of Health [17]. The system allows estimates regarding risk factors (expressed as DALYs per 100,000 people) to be acquired.

The acquired data regarding risk factors in 2011–2019—including metabolic risk factors, low physical activity, alcohol consumption, smoking, high BMI, air pollution and water pollution—were included in the generalized estimating equations model as predictors of one-year prevalence of melanoma in 2011–2019. Multicollinearity was assessed with the Variance Inflation Factor. Next, the predictions based on the model regarding years 2020–2021 were verified by comparison with the actual data regarding the one-year prevalence of melanoma in this period. Finally, predictions for the years 2023–2030 were calculated. A voivodeship in a specific year was the unit for the analyses performed.

The calculations were performed with the use of IBM SPSS Statistics 32.0 software.

## 3. Results

Table 1 depicts the one-year prevalence of melanoma for each voivodeship in 2011–2021 according to the Polish National Cancer Registry.

### Source: Polish National Cancer Registry

The number of melanoma cases in 2011–2021 increased in each voivodeship. On a national level, the increase was from 2571 cases to 3944 cases (53.4%). The highest prevalence of melanoma was detected in the Lesser Poland, Masovian, Pomeranian and Silesian voivodeships.

The burden of risk factors included in the analysis in 2011–2019, expressed as DALYs per 100,000 people according to the System Analysis and Implementation Database provided by the Polish Ministry of Health, is depicted in Table 2. This table depicts the average, the minimum, and the maximum value for DALYs regarding the risk factors in each voivodeship.

The highest DALYs were attributed to metabolic risk factors and the lowest to water pollution. The highest level of risk factors was detected in the Lublin and Silesian voivodeships and the lowest in the Subcarpathian and Pomeranian voivodeships.

Metabolic risk factors, low physical activity, and high BMI were expected to be highly correlated with each other. Therefore, firstly, multicollinearity was assessed by examining Variance Inflation Factor values. The values acquired are depicted in Table 3.

The values for three predictors, i.e., metabolic risk factors, low physical activity, and high BMI, exceeded the value of 10, indicating serious multicollinearity, which could inflate the variance of the coefficient estimates and lead to unreliable statistical inferences [18,19]. Collinearity diagnostics revealed that all three were highly correlated with each other. The proportion of variance for the same dimension was equal to 0.73 for metabolic risk factors and to 0.83 for high BMI. In another dimension, the proportion of variance was equal to 0.26 for metabolic risk factors and to 0.85 for low physical activity. To avoid unreliable statistical inferences, the three predictors were subjected to principal component analysis. The extracted common component was used as one of the predictors in further analysis. The factor loadings were equal to 0.98, 0.97 and 0.96 for high BMI, low physical activity and metabolic risk factors, respectively. The values of the factor loadings were very similar, suggesting equal impact of each indicator on the component value. This component accounted for 93.7% of the variance.

Data regarding UV radiation are not available for Polish voivodeships as a time series. However, the data from specific stations are. Therefore, the data regarding cloud-free DNA-damaging UV dose in kJ/m^2^ were acquired from the Thematic Emission Monitoring Integrated Services [20] for five measurement stations in Poland, i.e., Belsk, Legionowo, Leba, Warsaw and Zakopane. The annual average values are depicted in Table 4.

The values acquired from the stations were next used as estimates for the nearest voivodeships. Regarding Belsk, Legionowo and Warsaw, which are all in the same voivodeship, the arithmetic mean was used as an indicator for each year. The values from Belsk, Legionowo and Warsaw stations were used as estimators for the Lublin, Lodz, Masovian, Podlaskie, and Greater Poland voivodeships. The values from Leba station were used as estimators for the Kuyavian–Pomeranian, Lubusz, Pomeranian, Warmian–Masurian and West Pomeranian voivodeships. The values from Zakopane station were used as estimators for the Lower Silesia, Lesser Poland, Opole, Subcarpathian, Silesian, and Świętokrzyskie voivodeships.

The data regarding risk factors and prevalence of melanoma were analysed further using general estimating equations modelling with an autoregressive correlation matrix and Poisson loglinear model. We based the model on 11 years (2011–2021) × 16 voivodeships = 176 distinctive data points.

The model was based on the established risk factors with the addition of the following values for each voivodeship: population size, the proportion of inhabitants aged 60 or over, the proportion of males, and the values of cloud-free DNA-damaging UV doses. The results are depicted in Table 5.

The component consisting of metabolic risk factors, high BMI, and low physical activity was related to the prevalence of melanoma positively. Smoking and the prevalence of melanoma were associated positively as well. The proportion of inhabitants aged 60 or over was positively related to melanoma, and the proportion of males was negatively related to the prevalence of melanoma. Air pollution was negatively related to the prevalence of melanoma while controlling for other predictors.

For the purpose of prediction, only statistically significant predictors were used, i.e., the proportion of inhabitants aged 60 or over, the proportion of males, the component consisting of metabolic risk factors, high BMI and low physical activity, smoking, and air pollution. For the purpose of evaluating predictions, the values of risk factors’ DALY estimators were forecasted with the use of the linear trend. Predictions regarding the years 2020–2030 with 95% confidence intervals are depicted in Table 6.

The predictions for three voivodeships regarding 2020 and 2021 differed substantially from the actual data in terms of overestimation. This applies to Lublin, Lubusz and Opole voivodeships. The value of MAE (mean absolute error) was equal to 128.06; the value of RMSE (root mean squared error) was equal to 148.00. For further evaluation, we calculated the predictions using a simple baseline model, evaluating region-specific linear trends on the basis of the values from previous years. This yielded an MAE equal to 150.78, and the value of RMSE was equal to 171.45. Therefore, the current model revealed better accuracy.

## 4. Discussion

The analysis performed for the purpose of the current paper showed that, on the basis of statistical modelling, an increase regarding the prevalence of melanoma is predicted in all 16 Polish voivodeships. These predictions were based on five risk factors, specifically metabolic risk factors, low physical activity, alcohol consumption, smoking, high BMI, air pollution, and water pollution. However, metabolic risk factors, high BMI, and low physical activity were analysed as a combined component due to strong associations between these three risk factors.

This combined component was found to be associated positively with the prevalence of melanoma. Metabolic conditions encompassing obesity, insulin resistance, T2DM, and physical inactivity appear to collectively influence melanoma biology. Obesity-related dysfunctional adipose tissue engages in active crosstalk with melanoma cells through pro-inflammatory, pro-angiogenic, and lymphangiogenic factors [21,22]. Western nutrition, sedentary lifestyle, and obesity converge on oncogenic miR-21 signalling pathways that promote melanoma proliferation, evasion from apoptosis, angiogenesis, and metastasis [22]. Wang et al. propose a comprehensive framework categorizing metabolic conditions as a distinct category influencing melanoma initiation and progression [23].

When controlling for other risk factors included in the analysis, smoking was positively related to the prevalence of melanoma, which is consistent with some studies [11] but inconsistent with others [12].

Air pollution was negatively related to the prevalence of melanoma, which is inconsistent with other papers on the subject [13,24,25]. Specifically, particulate matter (PM), nitrogen oxides, and reactive oxygen species (ROS) induced by air pollution were mentioned as risk factors. However, statistical models provide estimates for individual predictors taking other predictors included in the analysis into account. As a consequence, a conclusion for each predictor should be made with the awareness that it is only valid when controlling for other predictors included in the model with a different set of predictors; conclusions may be different due to the relationships between predictors included and the strength of relationships between the predictors and the outcome analysed.

The proportion of males in the population was negatively related to the prevalence of melanoma. In national adult melanoma cohorts, multiple analyses consistently show higher incidence in females during the 20–39 and 40–64 age ranges, with a crossover or convergence toward male predominance at older ages in some populations, reflecting complex gender- and age-dependent risk profiles. Population-based analyses of young adults (20–30 s) frequently report more females diagnosed with melanoma than males in these age bands, while many European and North American datasets show increasing female predominance in younger ages and modest/perhaps male predominance later, though regional variability exists [26,27,28].

The proportion of inhabitants aged 60 or over was also positively related to the melanoma prevalence. Age is one of the most well-established factors associated with melanoma, as the risk of melanoma increases with age [29]. Therefore, this finding is consistent with the current scientific literature.

The main limitation of the current paper is that the predominant environmental risk factor for melanoma, i.e., the exposure to the ultraviolet radiation, was not included in the analysis. Unfortunately, the data regarding the history of UV radiation on the level of voivodeships are not available in Poland. The use of measurements from five stations as an approximate regional proxy yielded no statistically significant results. This definitely should not be interpreted as UV radiation having no effect on melanoma prevalence. Region-specific UV exposure, sunburn history, tanning behaviour, skin phototype, screening intensity, and dermatological access would be an appropriate set of predictors if the data were available. In the current analysis, measurements from just five stations were extrapolated to multiple voivodeships; historical cumulative exposure was not unavailable, and behavioural UV exposure was not measured. This limitation may introduce substantial residual confounding. The inability to adequately account for UV exposure severely limits interpretation of the observed associations between lifestyle factors and melanoma burden. A similar analysis performed on global data with UV radiations levels included could yield interesting results [30]. Another limitation is that the conclusions from the analysis based on large data units like voivodeships cannot be easily transferred to individuals. Both the predictors and the outcome were aggregated at the voivodeship level, which means the analysis was strictly ecological. Ecological associations cannot lead to individual-level etiologic inferences. The statistical model itself is also limited. It was based on 176 distinctive data points only. For future analysis, a model based on more data points, if data are available, would definitely be more robust, as the correlations between the values acquired for the same voivodeships could be taken into account in a hierarchical statistical analysis. Furthermore, for the majority of risk factors, DALYs were used rather than direct exposure measures. They may reflect exposure levels relevant to melanoma development but also the regional structure of morbidity, mortality, age composition, and socioeconomic status. DALYs reflect disease burden, mortality, disability, demographic structure and healthcare access. They are not direct measures of smoking prevalence, obesity prevalence, physical inactivity, or alcohol consumption. Consequently, the observed associations may reflect broader regional health differences rather than actual exposure levels. Direct age-standardized estimates of the prevalence regarding each risk factor would definitely be better and more valid predictors. However, these are not available on the voivodeship level in Poland.

However, on the level relevant to public health management, like the level of voivodeships, the analysis depicted in the current paper shows that an increase in melanoma can be expected in the coming years. Also, a hypothesis can be formulated: educating societies on the topic of the negative consequences of alcohol consumption, if effective, can change this expected trend. This hypothesis needs further verification due to the ecological design of the current study.

The predictions shown in the current paper are to be validated with the use of data available in the future. Currently, their status is exploratory.

## 5. Conclusions

This study demonstrates a consistent increase in melanoma prevalence across all Polish voivodeships between 2011 and 2021, with model-based forecasts indicating a continued upward trend in all 16 regions through to 2030.

The analysis confirms that selected environmental and lifestyle-related factors contribute to regional variation in melanoma prevalence. In particular, metabolic risk factors, high BMI, low physical activity, and smoking show a positive association with melanoma prevalence.

At the same time, the inverse association observed with air pollution suggests complex interdependencies and potential confounding effects. These findings should be interpreted cautiously, as regression-based estimates depend on the set of included predictors and do not necessarily reflect causal relationships.

The predictive models, while moderately explanatory, provide a useful tool for short-term forecasting at the population level. However, their accuracy is limited in some regions, and our results highlight the need for improved data input, particularly regarding ultraviolet radiation exposure, which remains a key but unmeasured determinant.

From a public health perspective, the projected increase in melanoma burden suggests further evaluations of interventions targeting the reduction of unhealthy lifestyle regarding diet, low physical activity and smoking. Additionally, the study confirms that analyses conducted at the voivodeship level are valuable for health system planning, although they should not be directly extrapolated to individual-level risk.

## Figures and Tables

**Table 1:** One-year prevalence of melanoma for each voivodeship.

	Voivodeship															
Year	1.	2.	3.	4.	5.	6.	7.	8.	9.	10.	11.	12.	13.	14.	15.	16.
2011	150	137	116	49	162	287	390	73	134	64	188	303	81	94	232	111
2012	185	135	120	77	248	358	395	72	168	71	224	339	95	95	256	161
2013	165	106	126	69	231	282	457	90	168	54	210	345	117	81	329	117
2014	187	160	146	75	196	315	471	71	178	71	213	337	118	84	308	131
2015	297	190	188	90	213	325	535	62	190	86	215	385	132	121	306	182
2016	257	140	186	85	269	387	563	84	175	98	223	394	125	117	285	170
2017	243	192	179	85	264	392	573	82	198	104	253	400	140	118	356	144
2018	246	185	194	60	261	343	496	90	245	109	229	428	141	148	364	142
2019	256	179	182	70	279	352	570	79	226	116	217	366	133	109	380	161
2020	243	169	133	47	225	260	531	65	152	88	135	361	117	107	302	184
2021	364	227	199	65	326	340	504	102	220	135	209	420	142	142	361	188

1. Lower Silesia; 2. Kuyavian–Pomeranian; 3. Lublin; 4. Lubusz; 5. Lodz; 6. Lesser Poland; 7. Masovian; 8. Opole; 9. Subcarpathian; 10. Podlaskie; 11. Pomeranian; 12. Silesian; 13. Świętokrzyskie; 14. Warmian–Masurian; 15. Greater Poland; 16. West Pomeranian.

**Table 2:** Share of risk factors in 2011–2019 in Poland.

Voivodeship	1.	2.	3.	4.	5.	6.	7.
Lower Silesia	9 247 [9 009; 9 551]	395 [380; 416]	2 847 [2 817; 2 895]	6 242 [6 122; 6 342]	4 229 [4 089; 4 436]	1 972 [1 860; 2 200]	40 [37; 41]
Kuyavian–Pomeranian	8 266 [7 985; 8 635]	321 [309; 342]	2 537 [2 515; 2 579]	6 140 [5 990; 6 322]	3 676 [3 549; 3 903]	1 630 [1 513; 1 853]	43 [40; 45]
Lublin	10 422 [10 099; 10 694]	409 [397; 426]	3 643 [3 552; 3 768]	6 659 [6 477; 6 847]	4 446 [4 306; 4 621]	2 465 [2 354; 2 774]	56 [53; 58]
Lubusz	8 882 [8 663; 9 179]	358 [352; 371]	2 642 [2 585; 2 774]	5 495 [5 379; 5 692]	3 759 [3 671; 3 929]	1 809 [1 703; 2 071]	45 [41; 47]
Lodz	8 771 [8 502; 9 115]	333 [322; 352]	2 725 [2 679; 2 774]	5 929 [5 767; 6 089]	3 949 [3 822; 4 167]	1 775 [1 692; 2 007]	39 [36; 41]
Lesser Poland	8 027 [7 828; 8 201]	347 [338; 361]	2 088 [2 035; 2 131]	4 870 [4 739; 5 027]	3 462 [3 372; 3 610]	1 980 [1 873; 2 172]	47 [44; 49]
Masovian	8 644 [8 429; 8 785]	358 [348; 372]	2 814 [2 753; 2 921]	5 336 [5 198; 5 511]	3 917 [3 815; 4 050]	1 779 [1 687; 2 018]	44 [41; 46]
Opole	9 811 [9 413; 10 307]	393 [373; 421]	2 397 [2 330; 2 425]	5 773 [5 528; 5 970]	4 300 [4 128; 4 579]	2 122 [2 031; 2 310]	47 [44; 49]
Subcarpathian	7 576 [7 256; 7 906]	303 [288; 322]	2 125 [2 088; 2 161]	4 366 [4 207; 4 510]	3 240 [3 100; 3 469]	1 574 [1 505; 1 717]	48 [44; 50]
Podlaskie	8 337 [8 107; 8 652]	313 [295; 330]	2 941 [2 883; 2 969]	4 786 [4 635; 4 909]	3 501 [3 323; 3 733]	1 449 [1 381; 1 597]	49 [46; 51]
Pomeranian	7 779 [7 535; 8 083]	298 [279; 315]	2 520 [2 481; 2 591]	5 247 [5 147; 5 347]	3 457 [3 255; 3 651]	1 195 [1 141; 1 327]	51 [47; 53]
Silesian	10 169 [9 907; 10 541]	405 [379; 432]	3 100 [3 028; 3 209]	5 786 [5 661; 5 930]	4 419 [4 252; 4 635]	2 488 [2 373; 2 675]	41 [37; 42]
Swietokrzyskie	9 547 [9 300; 9 840]	388 [376; 409]	2 623 [2 572; 2 683]	5 880 [5 727; 5 963]	3 997 [3 880; 4 201]	2 074 [1 984; 2 296]	40 [37; 42]
Warmian–Masurian	8 219 [7 876; 8 698]	311 [284; 336]	2 928 [2 862; 2 966]	5 882 [5 623; 6 128]	3 617 [3 389; 3 902]	1 492 [1 403; 1 659]	46 [43; 48]
Greater Poland	8 122 [7 901; 8 386]	323 [309; 340]	2 475 [2 445; 2 506]	5 450 [5 313; 5 539]	3 681 [3 567; 3 873]	1 674 [1 601; 1 857]	40 [37; 42]
West Pomeranian	8 477 [8 170; 8 829]	324 [312; 343]	2 666 [2 587; 2 748]	6 327 [6 105; 6 495]	3 810 [3 675; 4 026]	1 525 [1 445; 1 732]	42 [39; 43]

1. Metabolic risk factors; 2. low physical activity; 3. alcohol consumption; 4. smoking; 5. high BMI; 6. air pollution; 7. water pollution. Source. Calculations based on the System Analysis and Implementation Database provided by the Polish Ministry of Health.

**Table 3:** Variance Inflation Factors for analysed risk factors.

Risk Factor	*VIF*	Risk Factor	*VIF*
Metabolic risk factors	22.62	High BMI	21.22
Low physical activity	12.71	Air pollution	4.52
Alcohol consumption	2.84	Water pollution	4.57
Smoking	2.84	-	-

Source. Calculations based on the System Analysis and Implementation Database provided by the Polish Ministry of Health and Polish National Cancer Registry.

**Table 4:** Annual average values of cloud-free DNA-damaging UV doses from measurement stations in Poland.

	Station				
	Belsk	Legionowo	Warsaw	Leba	Zakopane
2011	0.804	0.782	0.793	0.681	0.944
2012	0.795	0.774	0.784	0.662	0.932
2013	0.732	0.715	0.723	0.631	0.839
2014	0.716	0.697	0.706	0.608	0.841
2015	0.726	0.705	0.716	0.605	0.856
2016	0.750	0.729	0.739	0.636	0.879
2017	0.775	0.754	0.764	0.653	0.909
2018	0.742	0.722	0.732	0.637	0.866
2019	0.770	0.750	0.760	0.646	0.900
2020	0.784	0.763	0.773	0.662	0.923
2021	0.755	0.736	0.745	0.641	0.878
2022	0.732	0.714	0.721	0.625	0.858
2023	0.771	0.747	0.757	0.640	0.911

**Table 5:** Results of generalized estimating equations analysis predicting the one-year prevalence of melanoma in a voivodeship on the basis of risk factors and population demographics.

Predictors	*IRR*	*p*
(Constant)	878,801.66 [4907.28; 157,376,966.74]	0.001
Metabolic risk factors	1.22	0.045
High BMI	[1.01; 1.48]	
Low physical activity		
Alcohol consumption	1.00 [0.99; 1.01]	0.351
Smoking	1.01 [1.01; 1.02]	0.049
Air pollution	0.999 [0.998; 0.999]	0.001
Water pollution	1.02 [0.99; 1.04]	0.203
DNA-damaging UV dose	2.26 [0.64; 7.66]	0.208
Age 60+	1.01 [1.00; 1.01]	0.029
Population size	1.00 [0.99; 1.01]	0.206
Male proportion	0.01 [0.01; 0.01]	0.001

*IRR*—incidence rate ratio with 95% confidence interval; *p*—statistical significance. Source. Calculations based on System Analysis and Implementation Database provided by the Polish Ministry of Health, Polish National Cancer Registry and Thematic Emission Monitoring Integrated Services.

**Table 6:** Predictions regarding prevalence of melanoma for 2020–2030 rounded to integers.

	Voivodeship															
Year	1.	2.	3.	4.	5.	6.	7.	8.	9.	10.	11.	12.	13.	14.	15.	16.
Actual data																
2020	243	169	133	47	225	260	531	65	152	88	135	361	117	107	302	184
2021	364	227	199	65	326	340	504	102	220	135	209	420	142	142	361	188
Predictions																
2020	356 270;469	258 192;347	358 261;490	226 164;311	257 195;338	133 84;210	222 157;315	310 210;458	128 82;200	199 139;283	215 159;290	295 186;467	271 194;379	258 196;339	190 138;261	284 212;380
2021	373 283;491	269 201;360	369 269;505	231 168;318	270 206;354	136 87;214	228 161;323	328 221;485	134 86;208	208 146;296	223 166;300	308 193;491	283 203;395	275 210;360	196 143;269	296 222;396
2022	390 296;515	280 210;374	380 278;520	237 173;325	284 217;370	140 89;219	234 165;331	346 233;514	140 90;216	217 153;309	232 173;311	322 200;517	295 212;412	293 225;383	203 149;277	310 232;414
2023	409 310;540	292 219;389	392 286;536	243 178;333	298 229;388	155 101;237	239 169;340	366 245;546	146 95;224	227 160;322	241 180;323	336 208;544	308 221;430	313 241;407	211 155;286	324 243;432
2024	428 323;568	304 229;404	404 294;553	249 182;341	313 241;407	146 94;228	245 172;349	386 258;580	152 99;233	238 168;337	250 187;335	351 215;573	322 231;449	334 257;434	218 161;295	339 254;452
2025	449 337;597	317 239;421	416 303;571	256 187;349	329 253;427	150 96;233	251 176;358	408 271;616	159 104;242	249 176;353	260 194;347	367 223;603	336 241;470	357 274;464	226 168;304	354 265;473
2026	470 352;629	331 250;438	429 311;590	262 192;358	345 266;448	154 99;239	258 180;368	431 284;656	166 109;252	260 184;369	270 202;361	383 230;636	351 251;491	381 292;496	234 174;314	370 276;496
2027	493 367;662	345 260;456	442 320;610	269 197;367	363 279;472	157 101;244	264 184;378	456 298;698	173 115;262	272 192;386	280 209;375	400 238;671	367 261;514	406 311;531	242 181;324	387 288;519
2028	516 382;698	359 271;476	455 328;630	276 202;377	381 292;496	161 104;250	271 188;389	482 312;743	181 120;273	285 200;405	291 217;390	418 246;708	383 272;539	433 330;570	251 188;335	404 300;545
2029	541 397;736	375 283;497	469 337;652	283 207;386	400 306;523	165 107;255	277 192;400	509 327;792	189 126;284	298 209;425	302 225;407	436 255;748	400 283;565	463 350;612	260 195;347	422 312;572
2030	566 413;777	391 294;518	483 346;675	290 212;397	420 320;552	183 121;278	284 196;411	538 342;845	197 131;296	312 218;446	314 233;424	456 263;791	417 294;593	494 370;658	269 202;359	441 325;600

1. Lower Silesia; 2. Kuyavian–Pomeranian; 3. Lublin; 4. Lubusz; 5. Lodz; 6. Lesser Poland; 7. Masovian; 8. Opole; 9. Subcarpathian; 10. Podlaskie; 11. Pomeranian; 12. Silesian; 13. Świętokrzyskie; 14. Warmian–Masurian; 15. Greater Poland; 16. West Pomeranian. Note. The values below point estimators, e.g., 220;328, are minimum lower and maximum upper bounds of 95% confidence intervals. Source. Calculations based on the System Analysis and Implementation Database provided by the Polish Ministry of Health and Polish National Cancer Registry.

## Data Availability

Data are contained within the article.
